# Being an autonomous person with chronic disease

**DOI:** 10.3325/cmj.2016.57.608

**Published:** 2016-12

**Authors:** Øystein Ringstad

**Affiliations:** Department for Health and Social Studies, Østfold University College, Halden, Norway oystein.ringstad@hiof.no

Respect for patient autonomy is a fundamental ethical principle in clinical health care. In most conceptions of patient autonomy, it is required that the patient considers information and knowledge that is relevant to the matter in question. Thus, the metaphor of navigating knowledge landscapes describes something that patients need to do in order to exercise their autonomy (1,2). This paper discusses how this “navigation” may be described from two different points of view of autonomy. In the well-known model of patients’ decisions about informed consent (3), autonomy is seen as a characteristic of decisions. However, autonomy can also be seen as a characteristic of persons. A concept called “Actual autonomy” is more relevant to contexts of an autonomous person with a chronic disease or disability (4). Here, decisions about informed consent are often less relevant.

## Autonomous decisions about informed consent

Today, most people associate patient autonomy with decisions about informed consent, which has gained status of the standard procedure for autonomous patient decision (3). However, informed consent is relevant primarily in situations where practitioners can provide recommendations about specific interventions together with relevant and reliable information. This means that the patient’s knowledge and understanding of the choice in question is largely determined by the recommendation and information received from the practitioner. We might say that the professional, evidence-based knowledge landscapes take precedence over the patient perspective where health care is provided as an intervention performed by the practitioner.

## Autonomous persons with chronic disease: actual autonomy

In cases where people live with chronic disease or disability, the situation is often quite different. Here, the question is rather what it means to be an autonomous person. A person’s life consists of more than making decisions. In-between the decisions, we live our lives more or less habitually, without thinking explicitly about the pros and cons of a specific matter. The typical clinical situation is when injury or disease becomes part of the person’s everyday life for a long time. Where medicine cannot offer a cure, autonomy becomes more about dealing with the disease than about making decisions on health care interventions.

Practitioners and researchers working with patient autonomy in long-term care have searched for other concepts of patient autonomy than informed consent (4-11). The American philosopher George Agich suggested the concept of “actual autonomy” (4). It was first presented in 1993 as a description of the autonomy of old people in long-term care, but it has also been applied to patients with chronic disease and disability (9-11).

Agich (4) asked how elderly people in long-term care actually exercised their autonomy. He found that the social world of everyday life was the relevant context for this discussion. Applied to people who experience lasting symptoms of a disease or injury, the immediate consequences are often that they cannot continue with the work-, family-, or friend-related activities that are important to them. Autonomy then becomes a question of retaining control over one’s daily life in spite of physical limitations and increased dependence on other people. However, at an underlying level, the person will sooner or later also need to redefine his or her identity according to the changed physical, practical, and social conditions. When former activities and roles that were important constituents of one’s self-concept cannot be continued, autonomy also becomes a question of finding new answers to questions such as “”Who am I?” and “What kind of person do I want to be?”.

In these situations, the patients’ personal experiences and perceptions concerning their everyday life with disease or disability become more important as sources of knowledge and understanding. Agich (4) found that patients’ life stories, or biographical narratives, were appropriate to view the meaning of actual autonomy. The concept of an autonomous person presupposes the personal identity and biography of a unique person. However, biographical narratives should not be understood only as detached reproductions of objective facts about the past of individuals. There are two important features of such narratives that are central in Agich’s analysis.

The first is that older people who tell their life stories “actively rearrange and reconstitute memories as a way of establishing location and direction in the (sometimes alien) present world of experience” (4). People also tell different stories about themselves under different circumstances and at different phases of life. Agich saw this as “an important manifestation of the actual autonomy of the elder to give meaning to her life and to make her present experiences meaningful” (4).

Furthermore, what people tell about their lives reveals what is important to them. It is not only a negotiation of meaning, but also a process of identification, where people tell what they value the most, who they want to be, and with what they identify.

The second important feature is that the person herself is not necessarily in a unique or privileged position to construct her own life story. Other persons who know somebody’s life story may contribute to the construction of a narrative. In Agich’s words: “Constructing a biography is, therefore, an important social action that occurs in long-term care as in other areas of social life” (4).

## Patients reconstructing their life stories and the physician’s contribution

How can the concept of “actual autonomy” be applied to the patient-physician relationship? In the light of this concept, respecting the autonomy of patients with chronic conditions does not solely mean obtaining their consent to medical interventions. It also means to respect and support the patient’s efforts to reconstruct their own life stories in order to create meaning and find out what to value and identify with in a life with chronic disease.

In the knowledge landscapes of most Western cultures, words like “development” and “human growth” usually mean an improvement that is orderly and predictable (4). When people experience a deterioration of their health and functional abilities with a corresponding dependence on others, this development does not fit with standard cultural perceptions of a happy and successful life. It rather counts as disorder and decay. However, such perceptions are matters of interpretation and part of the social construction of meaning. People in this situation may need to develop new perspectives on life that has taken an unfavorable and unpredictable course and new ways of understanding and telling the stories of their lives.

They may search for information and explanations from different sources, and not only those with proper quality assurance. In online and off-line media, they find stories about cases similar to their own, opinions about causes, new tests, treatments, and prognosis. Their search for information and knowledge and interpretation of what they find may be influenced by difficult emotions, such as fear, anger, shame, blame, and hopelessness. They need to regain their basic feelings of hope, belonging, and self-respect. Thus, the disease and corresponding negotiation of meaning can be seen as a context that has a great impact on how the information is obtained, understood, and incorporated in the patients’ stories. In the reconstructed narrative, the facts and knowledge will have to be incorporated in the context of the patient’s life with chronic disease (2). Where it comes to understanding of the knowledge about the disease, its causes, treatment options, prognosis and consequences for everyday life, patients who are otherwise known as rational and critical people may sometimes appear indiscriminate or fall victims of misinformation.

However, if health care practitioners do not acknowledge the patient’s search for new knowledge as an effort to create meaning, they may override and devaluate the patient’s efforts with correct, evidence-based professional knowledge and fail to show respect and provide support. Subsequently, constructing a biography is also an important social action and health care practitioners may consider to contribute to the patient’s narrative.

The physician and medical ethicist Howard Brody addressed this issue in an article “My story is broken, can you help me fix it?” (12). Brody argued that it was necessary to listen carefully to the patient’s own story in order to give information and views that the patient could recognize as being about *his* illness. Still, it was important that the physician’s views were biomedically sound and congruent with appropriate scientific thought. The physician’s contribution should facilitate “either the patient’s getting on with his life story or his modifying it as required by his illness” (12).

Respecting and supporting the patient does not necessarily mean to agree with or confirm different beliefs and perceptions the patient might adopt and incorporate in his or her narrative. It may be necessary to correct or supplement the patient’s understanding. However, the physician should endeavor to discuss such matters in a respectful way, seeing the patient’s views as an expression of her autonomy.
